# Correction: Dufvenberg et al. Six-Month Results on Treatment Adherence, Physical Activity, Spinal Appearance, Spinal Deformity, and Quality of Life in an Ongoing Randomised Trial on Conservative Treatment for Adolescent Idiopathic Scoliosis (CONTRAIS). *J. Clin. Med.* 2021, *10*, 4967

**DOI:** 10.3390/jcm12227079

**Published:** 2023-11-14

**Authors:** Marlene Dufvenberg, Elias Diarbakerli, Anastasios Charalampidis, Birgitta Öberg, Hans Tropp, Anna Aspberg Ahl, Hans Möller, Paul Gerdhem, Allan Abbott

**Affiliations:** 1 Department of Health, Medicine and Caring Sciences, Unit of Physiotherapy, Linköping University, SE 581 83 Linköping, Sweden; birgitta.oberg@liu.se (B.Ö.); allan.abbott@liu.se (A.A.); 2 Department of Clinical Science, Intervention and Technology (CLINTEC), Division of Orthopaedics and Biotechnology, Karolinska Institutet, SE 141 86 Stockholm, Sweden; elias.diarbakerli@sll.se (E.D.); anastasios.charalampidis@sll.se (A.C.); hans.moller@rkc.se (H.M.); paul.gerdhem@sll.se (P.G.); 3 Department of Reconstructive Orthopaedics, Karolinska University Hospital Huddinge, SE 141 86 Stockholm, Sweden; 4 Department of Biomedical and Clinical Sciences, Linköping University, SE 581 83 Linköping, Sweden; hans.tropp@regionostergotland.se; 5 Center for Medical Image Science and Visualization, Linköping University, SE 581 83 Linköping, Sweden; 6 Department of Orthopaedics, Linköping University Hospital, SE 581 83 Linköping, Sweden; 7 Department of Orthopaedics, Ryhov County Hospital, SE 551 85 Jönköping, Sweden; 8 Stockholm Center for Spine Surgery, SE 171 64 Stockholm, Sweden

## 1. Text Correction

In the original publication [1], there was a mistake in the abstract. “brace night shift (NB)” should be changed to “scoliosis night brace (NB)”.

A correction has been made to ***“1. Introduction”***, ***“Paragraph 6”***: “brace night shift (NB)” should be changed to “scoliosis night brace (NB)”.

A correction has been made to ***“2. Materials and Methods”***, ***“2.2. Interventions”***, ***“2.2.1. Hypercorrective Boston Brace Night Shift”***: The Title “Hypercorrective Boston Brace Night Shift” should be changed to “Hypercorrective Boston Scoliosis Night Brace”.

A correction has been made to ***“2. Materials and Methods”***, ***“2.2. Interventions”***, ***“2.2.1. Hypercorrective Boston Brace Night Shift”*:** “hypercorrective Boston brace night shift” should be changed to “hypercorrective Boston scoliosis night brace”.

## 2. Error in Table

In the original publication, there was a mistake in ***“Table 6”*** as published. Four data are missing in the NB group: Worse 7 (18), Unchanged 27 (71), Better 4 (10) and Indeterminable 0 (0). The corrected ***“Table 6”*** appears below.

**Table 6:** Health-related quality of life for the EQ-5D-Y dimensions, at baseline and 6 months, and changes in health state according to Paretian Classification of Health Change from baseline to 6 months.

EQ-5D-Y Dimensions	Mobility		Self-Care		Usual Activities		Pain/Discomfort		Anxiety/Depression	
	Baseline	6 Months	Baseline	6 Months	Baseline	6 Months	Baseline	6 Months	Baseline	6 Months
No problems										
NB-number (%)	44 (100)	39 (100)	44 (100)	38 (100)	42 (96)	38 (97)	34 (77)	30 (77)	28 (65)	25 (66)
SSE	43 (98)	39 (98)	44 (100)	40 (100)	41 (93)	37 (92)	33 (75)	26 (65)	31 (74)	29 (74)
PA	45 (100)	38 (100)	45 (100)	38 (100)	43 (96)	37 (97)	34 (76)	28 (74)	32 (71)	29 (76)
Moderate problems										
NB-number (%)	0 (0)	0 (0)	0 (0)	0 (0)	2 (4)	1 (3)	9 (20)	8 (20)	14 (33)	12 (32)
SSE	1 (2)	1 (2)	0 (0)	0 (0)	3 (7)	3 (8)	9 (20)	13 (32)	11 (26)	9 (23)
PA	0 (0)	0 (0)	0 (0)	0 (0)	2 (4)	1 (2)	10 (22)	9 (24)	12 (27)	8 (21)
Severe problems										
NB-number (%)	0 (0)	0 (0)	0 (0)	0 (0)	0 (0)	0 (0)	1 (2)	1 (3)	1 (2)	1 (3)
SSE	0 (0)	0 (0)	0 (0)	0 (0)	0 (0)	0 (0)	2 (4)	1 (2)	0 (0)	1 (3)
PA	0 (0)	0 (0)	0 (0)	0 (0)	0 (0)	0 (0)	1 (2)	1 (3)	1 (2)	1 (3)
Chi^2^ *p*-Value	0.662	> 0.999	NA	NA	0.898	0.617	>0.999	0.869	0.852	0.906
Paretian Classification of Health Change, baseline to 6 months	Worse ^1^		Unchanged ^2^		Better ^3^		Indeterminable ^4^		Chi^2^ *p*-Value	
NB *n* = 38-number (%)	7 (18)		27 (71)		4 (10)		0 (0)		0.448	
SSE *n* = 37-number (%)	8 (22)		22 (60)		6 (16)		1 (3)			
PA *n* = 38-number (%)	10 (26)		18 (47)		9 (24)		1 (3)			

EQ-5D-Y: EuroQol 5-Dimensions Youth; NB: hypercorrective Boston brace night shift; SSE: scoliosis-specific exercise; PA: adequate self-mediated physical activity; NA: not applicable—the distribution is a constant in self-care. Paretian Classification of Health Change: ^1^ The health state is worse in at least one dimension, and is no better in any other dimension; ^2^ the health state is exactly the same; ^3^ the health state is better in at least one dimension and is no worse in any other dimension; ^4^ the changes in health are “mixed”—better in one dimension, but worse in another.

## 3. Error in References

In the original publication, there was a mistake in ***“Reference 32”*** as published. “Boston Orthotics and Prosthetics. Boston Brace Night Shift Scoliosis Brace. Available online: https://www.bostonoandp.com/products/scoliosis-and-spine/boston-night-shift/ (accessed on 4 September 2020).” Should be changed to “Camp Scandinavia. Boston Skoliosis Night Brace. Available online: https://www.camp.se/produkter/ryggortoser/rigida/boston-skoliosis-night-brace-p28118 (accessed on 14 September 2021)”.

The authors apologize for any inconvenience caused and state that the scientific conclusions are unaffected. This correction was approved by the Academic Editor. The original publication has also been updated.
